# Endovascular management of a giant aortic arch aneurysm diverticulum: a case report

**DOI:** 10.1093/ehjcr/ytaf054

**Published:** 2025-02-05

**Authors:** Sherif Sultan, Yogesh Acharya, Riyad Ibrahim, Juan Carlos Parodi, William Wijns, Osama Soliman

**Affiliations:** Department of Vascular and Endovascular Surgery, Western Vascular Institute, University Hospital Galway, University of Galway, Newcastle Rd, Galway H91 YR71, Ireland; Department of Vascular and Endovascular Surgery, Galway Clinic, and Royal College of Surgeons in Ireland and University of Galway, Galway Affiliated Hospital, Doughiska Rd, Galway H91 HHT0, Ireland; Department of Vascular and Endovascular Surgery, Western Vascular Institute, University Hospital Galway, University of Galway, Newcastle Rd, Galway H91 YR71, Ireland; Department of Vascular and Endovascular Surgery, Western Vascular Institute, University Hospital Galway, University of Galway, Newcastle Rd, Galway H91 YR71, Ireland; Department of Vascular Surgery & Biomedical Engineering, Alma Mater, Trinidad Hospital, University of Buenos Aires, Calle Viamonte 430/444, Buenos Aires C1053ABH, Argentina; The Lambe Institute for Translational Medicine and Curam - Galway, University of Galway, Upper Newcastle, Galway H91 W2TY, Ireland; Precision Cardiovascular Medicine & Innovation Institute (PCMI), Cardiovascular Research Institute Dublin (CVRI Dublin), Mater Private Network & RCSI University of Medicine and Health Sciences, Eccles Street, Dublin D07 KWR1, Ireland

**Keywords:** Thoracic aortic diverticulum, Kommerell’s aneurysm, Hybrid approach, Carotid–carotid bypass, Endovascular aneurysm arch repair, COVID-19, Case report

## Abstract

**Background:**

Thoracic aortic diverticulum, or Kommerell’s aneurysm, is a developmental outpouching at the anteromedial aspect of the thoracic aorta, specifically at the site of the aortic isthmus, with an incidence of up to 9% in adults. It represents a notable anatomical variation that, over time, undergoes aneurysmal dilatation, posing the risk of rupture.

**Case summary:**

We present a 66-year-old male initially referred with a 7.35 cm giant thoracic aortic arch diverticulum (TAD). It was initially discovered incidentally as a 2.1 cm type 3 Salomonowitz; however, it subsequently increased to 7.36 cm following the third COVID-19 infection with cytokine storm. He underwent a right-to-left carotid necklace C-shaped configuration cross-over bypass using an 8 mm ringed Dacron silver graft. Six weeks post-bypass, the patient underwent a successful repair of TAD using the NEXUS-Endospan-Artivion system (Artivion™, GA 30144, USA), employing a double inner branch to the innominate and left subclavian artery through a single groin approach. Postoperatively, he recovered fully without any neurological or cardiovascular issues with no signs of endoleaks, graft migration, or separation.

**Discussion:**

The saccular nature of the aneurysm at the arch reveals a distinctive set of challenges, mainly the low wall shear stress, which exposes their malignant potential, emphasizing the crucial need for intervention, especially when surpassing the critical 30 mm threshold. This premiere marks a significant milestone by introducing the ‘IDEALIST’ Artivion/Endospan Nexus, which pioneers a total endovascular approach post-CE marking, ushering in a new era in aortic arch interventions.

**Learning points**

- Thoracic aortic diverticulum, or Kommerell’s aneurysm, is a developmental outpouching of the thoracic aorta that, over time, undergoes aneurysmal dilatation, posing the risk of rupture.
- The saccular nature of the aneurysm at the arch reveals a distinctive set of challenges, mainly the low wall shear stress, which exposes their malignant potential, emphasizing the crucial need for intervention, especially when surpassing the critical 30 mm threshold.
- The integration of ‘single groin dual chamber double inner branch endograft technology’ not only addresses the challenges posed by these unique vascular conditions but also heralds a new era in the management of arch aneurysms.

## Primary specialties involved other than cardiology

Vascular surgery, cardiothoracic surgery, anaesthesia, and intensive care

## Introduction

Navigating the complexities of aortic arch aneurysms has long been a formidable challenge in vascular surgery. The aortic arch, marking the culmination of the aorta’s journey, has historically posed risks that necessitate careful consideration.

**Figure 1:**
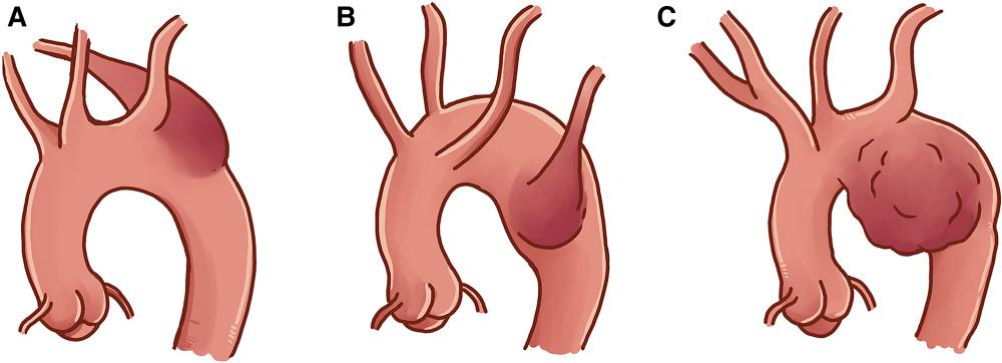
Diagram showing the Salomonowitz classification of aortic diverticula. (*A*) Type 1 diverticulum associated with the left so-called normal aortic arch and aberrant right subclavian artery. They are generally conical in shape. (*B*) Type 2 diverticulum in right anomalous aortic arch with aberrant left subclavian artery. They are larger and more rounded in configuration. (*C*) Type 3 diverticulum arising from the isthmus, the ductal zone of the thoracic aorta, not associated with the subclavian artery, best described as a non-Kommerell or ductal diverticulum.

We present a type III Salomonowitz of aortic ductal diverticula originating from the isthmus and ductal zone of the thoracic aorta, independent of the subclavian artery^1^ (*Figure 1*). Ductus arteriosus connects the aortic arch and pulmonary artery, closing after birth to form the ligamentum arteriosum. A focal bulge may persist, forming the aortic ductus diverticulum.^2^

## Summary figure

**Table:** 

July 2013	Incidental finding of 2.1 cm type 3 Salomonowitz while investigating chest pain and shortness of breath.
February 2015	Computed tomography angiography (CTA) scans revealed that the thoracic aortic arch diverticulum (TAD) expanded to 2.9 cm.
November 2016	CTA documented 3.1 cm TAD.
March 2020	TAD expanded to 5.6 cm after the patient suffered from the coronavirus disease 2019 (COVID-19) infection.
August 2022	Patient suffered from second COVID-19 infection. A follow-up CTA confirmed an alarming expansion of TAD to 6.5 cm following unexplained hoarseness of voice and dysphagia.
January 2023	A repeat CTA after the third COVID-19 infection confirmed a significant enlargement of TAD to 7.36 cm.
March 2023 (Day 0)	Patient underwent a right-to-left carotid necklace C-shaped configuration cross-over bypass using an 8 mm ringed Dacron silver graft.
April 2023 (Day 42)	Patient underwent a successful repair of TAD using the NEXUS-Endospan-Artivion system (Artivion™, GA 30144, USA), employing a double inner branch to the innominate and left subclavian artery through a single groin approach.
June 2023 (Day 84)	Follow-up CTA at 6 weeks demonstrated stable arch grafts with no signs of endoleaks, graft migration, or separation. Carotid and subclavian duplex scans indicated normal flow velocities on all the great vessels of the head and neck.
October 2023 (Day 180)	Follow-up CTA at 6 months showed that TAD shrank to 6.3 cm with total aortic remodelling without stent graft–induced new entry (SINE).
April 2024 (Day 360)	Follow-up CTA at 12 months showed that TAD shrank to 5.8 cm with normal aortic contour without SINE.

## Case presentation

**Figure 2:**
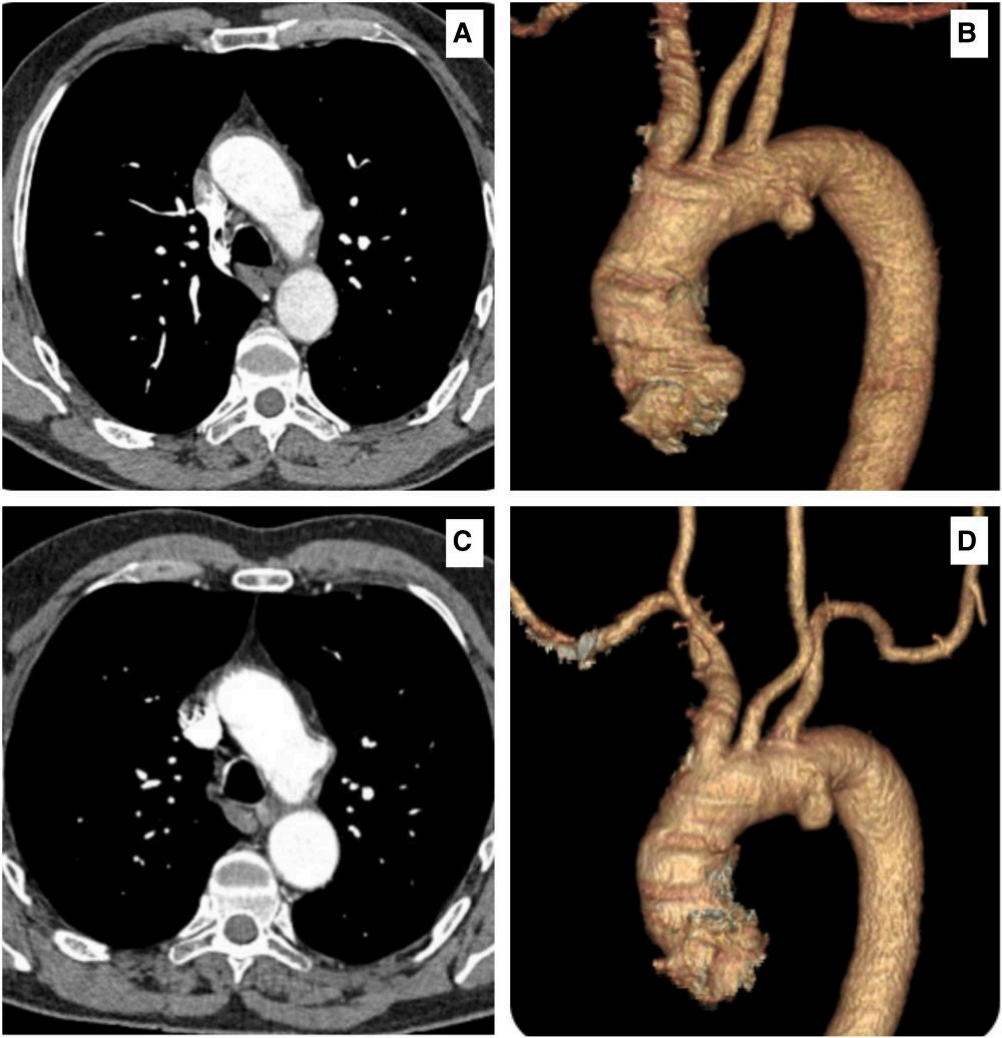
Computed tomography angiography scans and the 3D reconstructions, showing the following: (*A*, *B*) in July 2013, there was a 2.1 cm thoracic aortic arch diverticulum at the inner concavity of the aortic arch. (*C*, *D*) A 2.9 cm thoracic aortic arch diverticulum at the inner concavity of the aortic arch in February 2015.

We present a 66-year-old male initially referred with a 7.35 cm giant TAD. It was discovered incidentally in July 2013 while investigating chest pain and shortness of breath, revealing a 2.1 cm type 3 Salomonowitz initially (*Figure 2A* and *B*).

**Figure 3:**
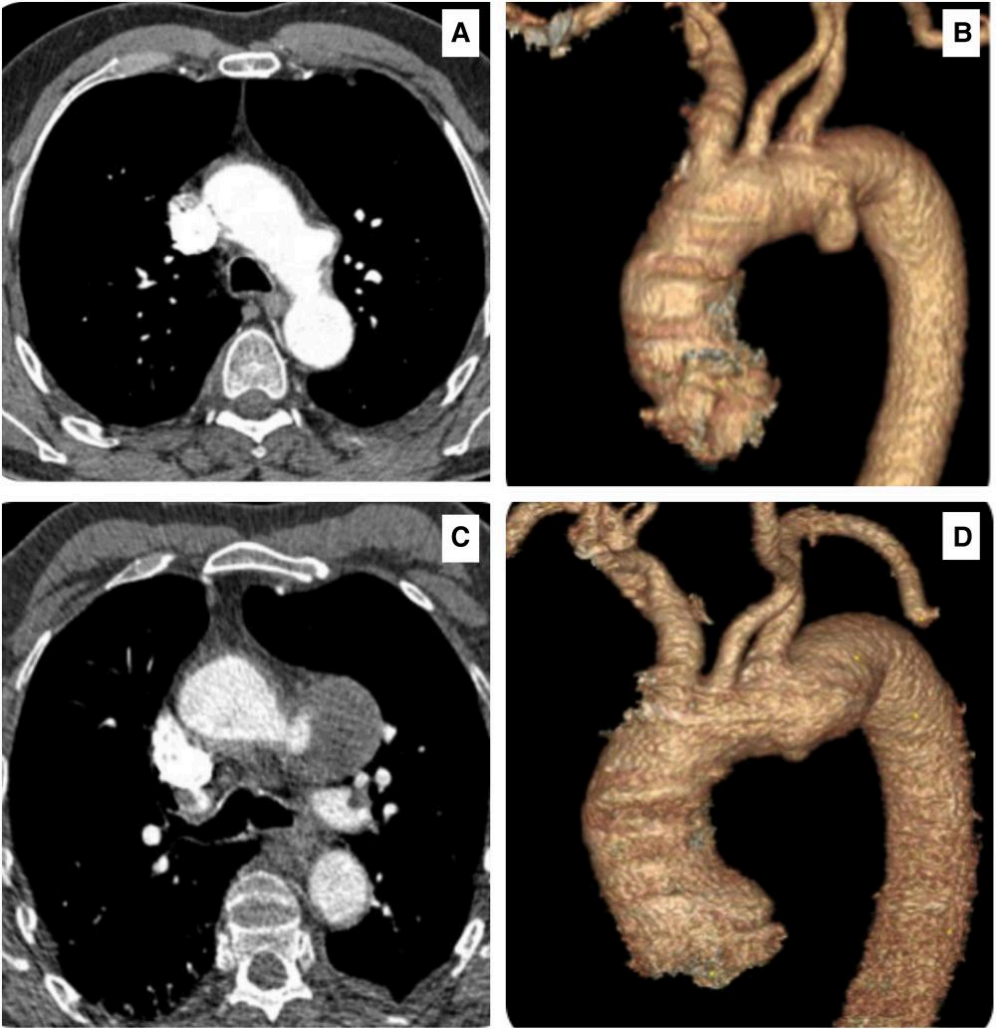
Computed tomography angiography scans and the 3D reconstructions, showing the following: (*A*, *B*) in November 2016, there was a 3.1 cm thoracic aortic arch diverticulum at the inner concavity of the aortic arch. (*C*, *D*) A 5.6 cm thoracic aortic arch diverticulum at the inner concavity of the aortic arch accelerated growth post-COVID-19 in March 2020.

In February 2015, the patient suffered from a bilateral pulmonary embolism, and subsequent CTA scans revealed that the TAD expanded to 2.9 cm (*Figure 2C* and *D*). A CTA in November 2016 documented 3.1 cm TAD (*Figure 3A* and *B*). A CTA in November 2016 documented a 3.1 cm TAD (*Figure 3A* and *B*). In January 2020, he contracted the COVID-19. His subsequent CTA in March 2020 showed that the TAD had expanded to 5.6 cm (*Figure 3C* and *D*).

**Figure 4:**
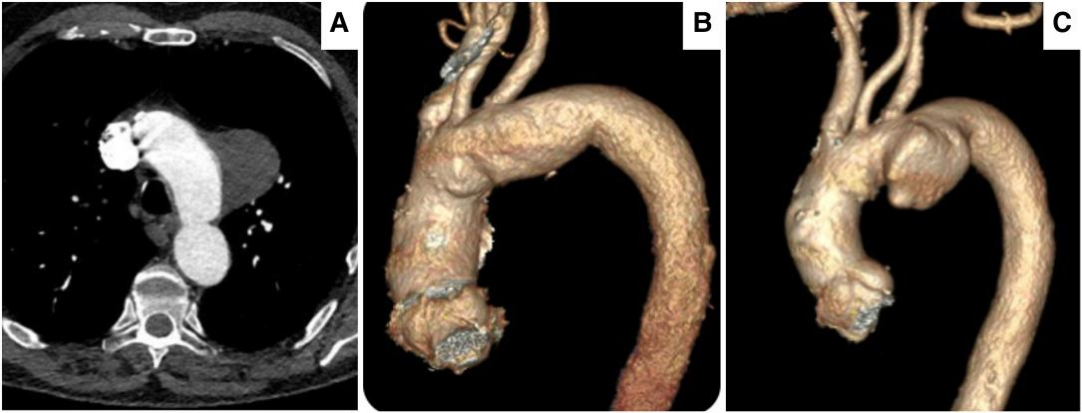
Computed tomography angiography scans and the 3D reconstructions, showing the following: (*A*, *B*) a 6.5 cm thoracic aortic arch diverticulum at the inner concavity of the aortic arch with accelerated growth post-second COVID-19 infection in August 2022. (*C*) A 7.34 cm thoracic aortic arch diverticulum at the inner concavity of the aortic arch with accelerated growth post-third COVID-19 infection in January 2023.

In late 2022, the emergence of unexplained hoarseness of voice and dysphagia, initially attributed to a second COVID-19 infection, prompted further investigation. A follow-up CTA confirmed an alarming expansion of TAD to 6.5 cm (*Figure 4A* and *B*).

The patient’s medical history included hypertension, emphysema, dyslipidaemia, hypothyroidism, gastroesophageal reflux disease, a 30-pack-year smoking history, and a background of recurrent deep vein thrombosis and pulmonary embolism. Notably, the patient was non-compliant with anticoagulation or antiplatelet medications.

On admission, his blood pressure was 160/88 mmHg, heart rate 78 b.p.m., respiratory rate 16 breaths/min, oxygen saturation 98% in room air, and temperature 36.5°C. His cardiovascular, respiratory, and gastrointestinal system examinations were otherwise unremarkable.

His total cholesterol was 5.18 mmol/L (normal: 3–5 mmol/L) with normal haemoglobin, white cell count, platelets, calcium, renal and liver function, D-dimer, and clotting profile.

Despite blood parameters remaining within normal limits, a repeat CTA after the third COVID-19 infection in January 2023 confirmed a significant enlargement of TAD to 7.36 cm (*Figure 4C*).

The indications for repair encompass various factors, including the saccular nature of the aneurysm at 7.36 cm with rapid expansion exceeding 5 mm per year with hoarseness of voice and recurrent lower respiratory lung infection with dysphagia.

The patient was considered for open aortic surgery for aortic arch treatment but was deemed unfit due to a recent pulmonary embolism, COVID-19 infection, and overall high surgical risk. To minimize the risk of micro- and macro-embolization, we avoided using the carotid arteries for access.

After consulting with the cardiothoracic surgeon, we opted for an endovascular approach using the NEXUS-Endospan-Artivion system (Artivion™, GA 30144, USA). To facilitate this safely, we performed a right-to-left common carotid cross-over bypass, allowing all wires and stents to be introduced through the subclavian arteries. This approach enabled us to treat the brachiocephalic and left subclavian arteries as branches of the aortic arch graft without direct carotid cannulation.

**Figure 5:**
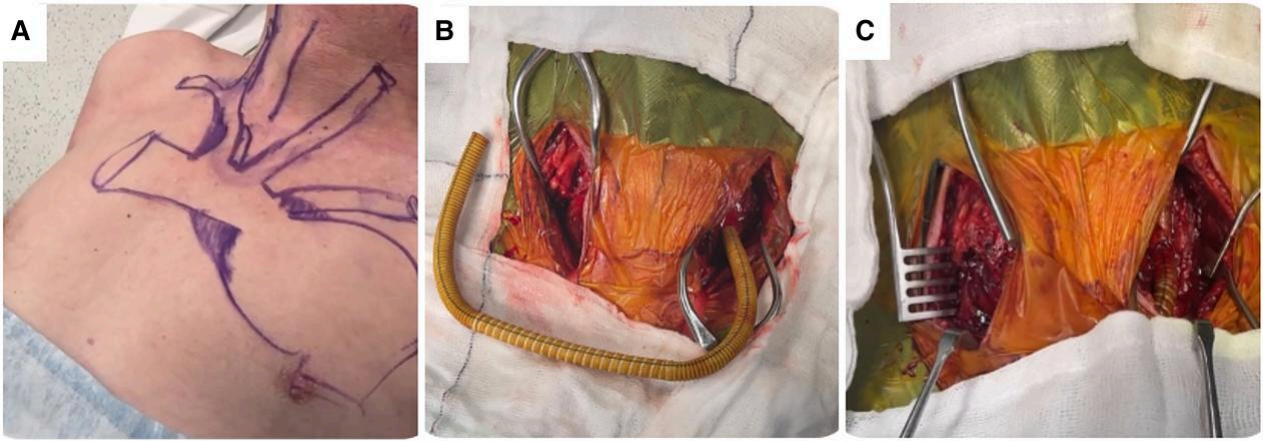
Right carotid to left carotid cross-over bypass by silver 8 mm ringed silver Dacron graft in a necklace C-shaped configuration in March 2023. (*A*) Depiction of the surface anatomy of the aortic arch and the right to left carotid where the cross-over will occur. (*B*) An 8 mm silver Dacron graft with rings for reinforcement to prevent any compression during the neck movements. The graft will be tunnelled subcutaneously in a necklace fashion. (*C*) Necklace C-shaped configuration post right to left carotid bypass done into the proximal common carotid.

Subsequently, the patient underwent a right-to-left carotid necklace cross-over bypass using an 8 mm ringed Dacron silver graft in March 2023 (*Figure 5A–C*). Carotid artery cannulation can cause intimal damage, leading to significant complications such as carotid artery dissection and bleeding with micro- and macro-embolization. To minimize these risks, we prefer using the subclavian artery for access during endovascular arch repair. This helps to reduce the likelihood of carotid injury, particularly during wire manipulation and other procedural manoeuvres. As a result, carotid access is strictly avoided unless the subclavian artery is occluded, ensuring a safer outcome for the patient.

**Figure 6:**
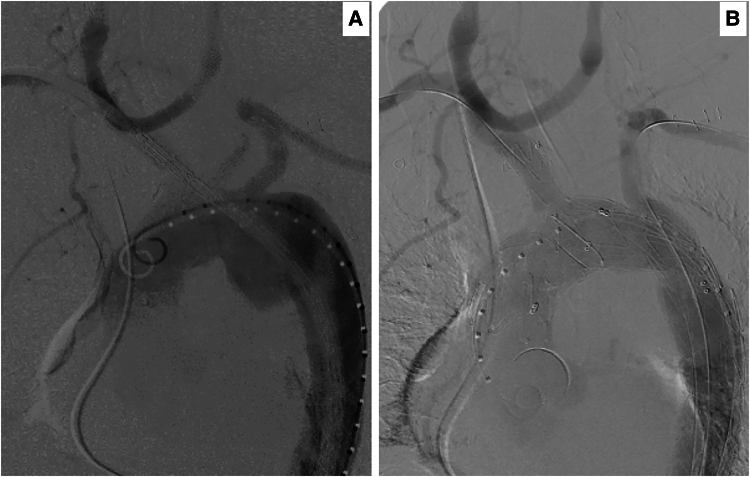
Digital subtraction angiography. (*A*) Stage 1, before deployment in a brachiocephalic arch. (*B*) Stage 2, post total NEXUS stent (Artivion™, GA 30144, USA) deployment with all great vessels of head and neck fully revascularized.

**Figure 7:**
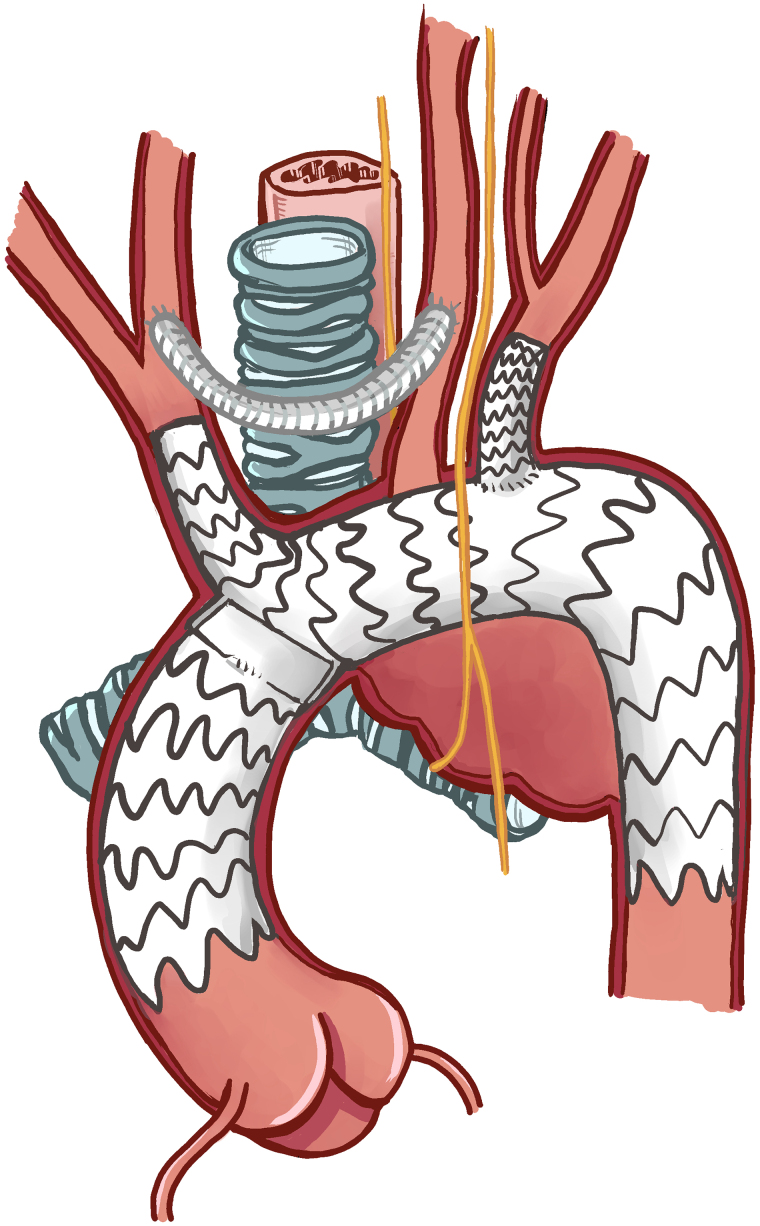
Diagram depicting the NEXUS aortic arch stent (Artivion™, GA 30144, USA) with right carotid to left carotid cross-over bypass by an 8 mm ringed silver Dacron graft in a necklace C-shaped configuration.

Six weeks post-bypass, the patient underwent a successful repair of TAD using the NEXUS-Endospan-Artivion system (Artivion™, GA 30144, USA), employing a double inner branch to the innominate and left subclavian artery through a single groin approach under controlled ventricular fibrillation (*Figures 6A* and *B* and *7*). This procedure required two bilateral proximal axillary artery cutdowns with a bilateral 9 French sheath and a right groin cutdown with 10 French sheaths replaced by 26/60 cm dry seal Gore (WL Gore & Associates, Inc., Flagstaff, Arizona, USA). Next, a 400 cm wire was delivered from the right axillary artery through the innominate branch to the groin, similar to a railway. Care was taken to avoid excessive rotation or tension on the left subclavian artery wire while crossing the arch, which was then replaced with a 400 cm Terumo wire. We then deployed the innominate branch, fully advancing the stent caudally towards the heart while inducing fibrillation to prevent stent migration. As the stent was positioned to face downwards, we deployed the entire graft. Once this was done, the delivery system for the first part of the graft was removed, and post-deployment ballooning of the graft was performed. The proximal part of the ascending graft was loaded and deployed 1.5 cm above the coronary ostium while fibrillation was maintained. Finally, the VBX stent was deployed through the left subclavian artery, with half positioned into the inner branch tunnel and the other half proximal to the vertebral artery.

We avoided adding an occlusion device to the left common carotid artery, as the aortic arch stent would occlude its ostium, reducing the hardware in the proximal left common carotid artery and minimizing infection risk. The total aortic arch endovascular treatment was successfully concluded by occluding the ostium of the left carotid artery without compromising blood flow, which was maintained through the right-to-left cross-over bypass.

**Figure 8:**
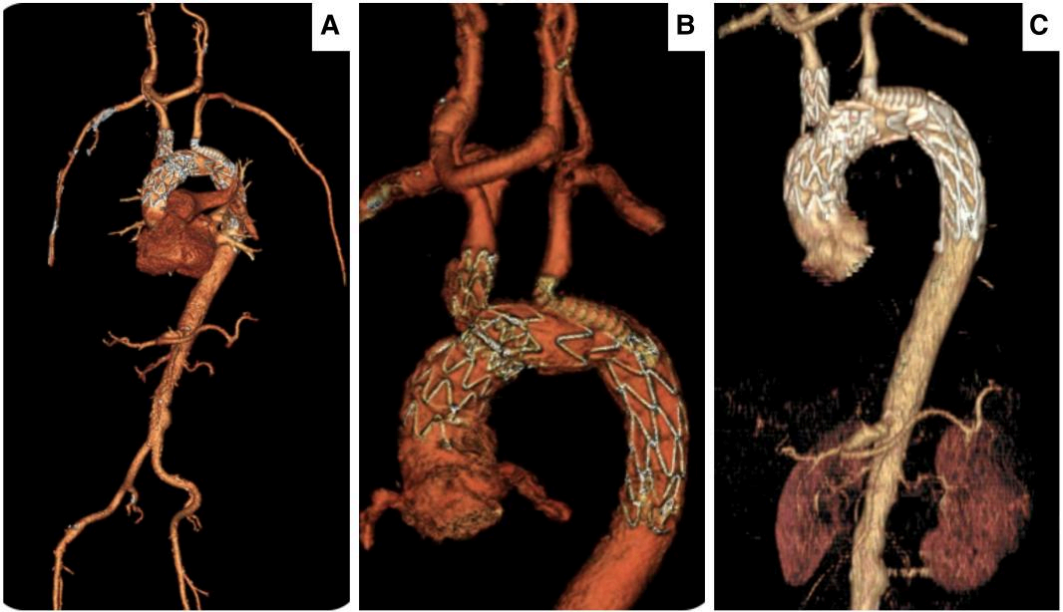
Post-operative 3D reconstructions of the computed tomography angiography scans, with no evidence of graft migration, twist, or endoleaks. (*A*) At 6-week follow-up, all the great vessels of the head and neck were patent. (*B*) At 6-month follow-up, the size of the aneurysm was 6.3 cm. (*C*) At 1-year follow-up, the size of the aneurysm was 5.8 cm.

There were no peri-operative complications, and he was asymptomatic and mobilizing well without functional limitations. Subsequent follow-ups at 6 weeks, 6 months, and 12 months demonstrated stable arch grafts with no signs of endoleaks, graft migration, or separation (*Figure 8A–C*). Carotid and subclavian duplex scans indicated normal flow velocities with shrinkage of TAD to 5.8 cm.

The patient has been on an indefinite regimen of direct oral anticoagulants (DOACs), apixaban (2.5 mg once daily), and antiplatelet, clopidogrel (75 mg once daily), given his previous history of DVT/PE and recent carotid artery bypass with subclavian stenting, contributing to a comprehensive and successful management strategy for this complex case.

## Discussion

The aortic arch is the final frontier for endovascular treatment, presenting unique anatomic and haemodynamic challenges that complicate the pursuit of safe and durable repairs. Limited data and heterogeneity across studies contribute to uncertainties in outcomes. High-volume centres, renowned for aortic expertise, consistently demonstrate improved outcomes in open aortic arch repair, serving as the benchmark against which hybrid repair is evaluated in both short- and long-term perspectives.

The arch’s distinctive anatomic and haemodynamic features, marked by its curvature and heightened pulsatility, create dynamic strain, posing complex challenges in TAD management.^3^ The four great head and neck vessels preside over concerns related to cerebrovascular perfusion, landing zone limitations, athero-emboli, vascular injury, and retrograde type A dissection. Deployment challenges persist, encompassing trackability, conformity, endograft apposition, seal, migration, kinking, fracture, and the potential for the ominous ‘windsock’ movement before fixation, highlighting the intricacies faced by even experienced interventionalists.^4,5^

Kommerell’s diverticulum, stemming from incomplete regression of the embryonic dorsal aorta, often evolves into an aneurysm, typically discovered post-dissection or rupture. This tissue’s vulnerability emphasizes the intricacies involved in its management.^6^

The Sultan group^7,8^ had outlined that the absence of comparative randomized clinical trials and a lack of level-one evidence in aortic arch aneurysm management make drawing parallels between open, hybrid, and total endovascular techniques inherently unfair. The fundamental differences in patient populations treated with these varied approaches and the need for an individualized strategy underscore the challenge of making direct comparisons and emphasize the importance of tailoring interventions for optimal results.^7,8^

The saccular nature of Kommerell’s diverticulum at the arch leads to a permanent dilation without proportional elongation, characterized by low wall shear stress (WSS), regardless of diameter. Those along the inner curvature tend to have a higher and variable sac depth/neck width ratio. When this ratio exceeds 0.8, the WSS remains low, irrespective of diameter, potentially explaining their malignant behaviour and the catastrophic rupture.^9–11^ Low WSS initiates and exacerbates atherosclerosis by influencing endothelial cell function. Maintaining an adequate level of WSS (>1.5 Pa/m²) is athero-protective, while low WSS (<0.4 Pa/m²) triggers a shift in endothelial phenotype towards atherogenic conditions.^9^ In aneurysms, low-velocity vortex flow decreases WSS,^10^ activating matrix metalloproteinase and contributing to vessel wall weakening and aneurysm growth.^11^ Notably, once an aneurysm forms, especially in the case of arch saccular aneurysms, low WSS perpetuates a vicious cycle, promoting further aneurysm growth. Recognizing the role of WSS underscores the importance of addressing saccular aneurysms, particularly when they surpass the critical threshold of 30 mm.^9–11^

The ‘NEXUS Aortic Arch Stent Graft System’ (Artivion™, GA 30144, USA), designed to address the aortic arch’s anatomy and haemodynamic challenges, is a newer endograft solution to address these complex arch pathologies. It exhibited a 30-day mortality rate of 7.1%, while the non-disabling stroke rate was 3.6%, resulting in a combined mortality and stroke rate of 10.7%.^12^ Over 1 year, the combined mortality and stroke rate increased to 17.8%, with 10.7% of patients experiencing device-related unplanned re-interventions.

Traditionally, minimally invasive procedures in zone 0 arch and branched endografts have been the primary endovascular alternatives to the frozen elephant trunk technique. However, the NEXUS double-branched endograft (Artivion™, GA 30144, USA), characterized by its bimodular design, introduces innovation with two inner branched arches and an anatomically pre-shaped ascending aorta module. The branch to the brachiocephalic trunk enables precise deployment before completing the aortic arch and descending aorta portion. Notably, the left subclavian branch enhances caudal stability without manipulating the carotid arteries, addressing prior concerns.

The dual-branched endograft system and advanced catheterization techniques represent a significant advancement in the treatment of aortic arch pathologies. By enabling a fully endovascular approach and reducing the need for open-heart surgery, this method allows for more precise and safer procedures.

Two significant concerns surrounding single-branched devices were the need for zone I debranching and reliance on a single feeder branch. These concerns were mitigated through a right-to-left carotid bypass, avoiding the reduction of blood flow and potential patency issues.^13^

The multicentre trial by D'Onofrio *et al.*^14^ provides a 3-year follow-up on 28 high-risk patients treated with the Nexus device, reporting a low peri-operative stroke rate of 7% and a lack of strokes during follow-up. Moreover, only 11% experienced Nexus-related re-interventions, with no type Ia endoleaks observed and type III endoleaks identified in only 4%.

The procedural success without stroke, as reported by Planer *et al.*,^12^ stands at 100%, attributing it to reduced wire manipulations during Nexus implantation, a decreased device profile to 20 French, and an advanced de-airing sequence with two flush ports in the deployment system. These factors collectively contribute to a significant advantage in preventing air embolism, marking a positive stride in the safety and efficacy of aortic arch interventions.

Ductus diverticulum aneurysms pose a rupture risk at 30 mm or larger, with higher catastrophic potential than saccular atherosclerotic aneurysms.^2^ The adult form becomes symptomatic late, presenting as a thoracic mass with symptoms like dyspnoea, cough, chest pain, and hoarseness due to left vocal cord paralysis.^15–18^

Since Pontone *et al.*^16^ described the first giant ductus arteriosus aneurysm (DAA) in 2010, a few other cases have been reported in English literature. Cardiovascular specialists recommend using ‘Kommerell aneurysm’ instead of ‘diverticulum’.^17^ We report our second case of giant symptomatic 7.34 cm TAD.^19^ Our first patient presented with voice hoarseness and recurrent respiratory infections. Direct laryngoscope revealed left recurrent laryngeal nerve palsy, consistent with Ortner’s cardiological syndrome.^18^ The natural course is considered grim, with a fragile aneurysmal wall constantly at rupture risk, leading to premature death without intervention.

The TAD stems from abnormal embryological remnants, classified into type I, II, and III Kommerell’s diverticulum. A Kommerell’s aneurysm is a dilatation of Kommerell’s diverticulum, with an average size of 50.7 ± 7.1 mm, found in 3%–8% of Kommerell’s diverticulum.^19,20^ A Kommerell’s aneurysm should be defined when Kommerell’s diverticulum exceeds 30 mm, as it poses a higher risk of rupture with distinct histological features of medial cystic necrosis.^19,20^

Structural alterations in Kommerell’s aneurysm may contribute to fatal complications, including distal embolization, compression, dissection, and rupture.^19,20^

Our patient experienced three bouts of COVID-19 infection, leading to hospitalization twice and necessitating IV steroid treatment following protocol. The severity of the COVID-19 infection induced a cytokine storm, marked by elevated levels of interleukin-1β, interleukin-6, IP-10, tumour necrosis factor, interferon-γ, macrophage inflammatory proteins 1α and 1β, which are known to correlate with disease severity and outcomes. These cytokines trigger the activation of metalloproteinases, enzymes capable of degrading collagen and elastin, resulting in aortic wall degeneration.^21^ Bozzani *et al.*^22^ reported a 9.7-fold increased likelihood of rapid growth of aortic aneurysms in COVID-19 patients. In our case, the acceleration in growth, as evidenced by the increase from 3.1 to 7.36, contributed to the development of giant TAD.

Our experience shows that the transformative impact of the ‘IDEALIST’ system extends beyond mere procedural innovation. It signifies a paradigm shift, eliminating the need for open-heart surgery and introducing a minimally invasive alternative. The preparatory debranching procedure, including a carotid–carotid cross-over bypass, sets the stage for evaluating safety and performance over a compelling 1-year period.

Beyond conventional approaches, the ‘idealist’ system integrates advanced catheterization techniques, state-of-the-art materials, and the power of artificial intelligence and machine learning in pre-operative and intraoperative planning. This synergy of cutting-edge technology is a beacon of precision, safety, and improved patient outcomes in the challenging landscape of aortic arch interventions.

## Conclusion

Navigating the complexities of TAD and its associated saccular aneurysms requires a proactive approach guided by the evolving landscape of endovascular solutions. Nexus technology emerges as a transformative endovascular option, poised to redefine our approach to arch aneurysms. Embracing this innovation is pivotal for optimizing patient care, and its impact promises to reshape the landscape of vascular interventions for years to come.

## Data Availability

The data underlying this article are available in the article and in its online Supplementary material.

## Lead author biography

Prof Sherif Sultan is a pioneering vascular/endovascular surgeon and the founder of the Western Vascular Institute, Ireland, a charitable research foundation committed to vascular research, technical innovation, and education. He is the current president of the International Society of Vascular Surgery and the American Society of Angiology, Irish chapter. He had pioneered the techniques of sub-intimal angioplasty, DRESS technique for complex EVAR, kinetic elephant trunk for pan-aortic dissection, TIGER protocol for infra-diaphragmatic aortic dissection, triple neuroprotection for patients with acute vascular stroke post the 24-h opportunity window, and Art-assist for critical limb ischaemia patients.

**Consent:** The authors confirm that written consent for submission and publication of this case report, including images and associated text, has been obtained from the patient in line with COPE guidance.

**Funding:** None declared.
